# Biomechanical analysis of biodegradable magnesium, zinc, and polylactide pins for fixation of radial head fractures

**DOI:** 10.1186/s13018-025-06346-2

**Published:** 2025-10-08

**Authors:** Julian P. Maier, Jonas Eck, Benjamin Erdle, Nils Mühlenfeld, Michael Seidenstuecker, Kilian Reising, Hagen Schmal, Ferdinand C. Wagner

**Affiliations:** 1 Department of Orthopedics and Trauma Surgery, University Medical Center Freiburg, Freiburg, Germany; 2 G.E.R.N Tissue Replacement, Regeneration & Neogenesis, Department of Orthopedics and Trauma Surgery, University Medical Center Freiburg, Freiburg, Germany; 3 Faculty of Medicine, University of Freiburg, Freiburg, Germany; 4 Department of Orthopedic Surgery, University Hospital Odense, Odense, Denmark

**Keywords:** Radial head fracture, Mason type II, Biodegradable implants, Zinc pins, Magnesium pins, Polylactide pins, Biomechanics

## Abstract

**Background:**

Biodegradable implants have raised constant interest for fixation of displaced radial head fractures due to avoiding implant removal and minimizing cartilage damage. Polylactide pins (PP) are frequently used in clinical practice, but inferior mechanical properties showed higher rates of secondary dislocation compared to metal implants. Magnesium pins (MP) provide superior stability but exhibit inconsistent resorption and relevant hydrogen gas formation. Recently, zinc pins (ZP) have emerged as a promising alternative, offering comparable mechanical strength with favourable biocompatibility. Since these implants have not been tested for specific fracture fixation, this study aims to evaluate their applicability in a validated Mason type II radial head fracture model.

**Methods:**

Standardized Mason type II fractures were conducted in biomechanically validated composite radii, and fixed by using either two 2.0 mm MPs, ZPs, or PPs. Biomechanical testing included 10 cycles of transverse loading, 1,000 cycles of axial loading (15–50 N at 0.1 Hz), and load-to-failure testing (2 N/sec). Stability was assessed by stiffness (kN/mm) under axial and transverse loading, fracture displacement (mm) after 1,000 cycles, and failure load (N) at dislocation ≥ 2 mm.

**Results:**

MPs demonstrated the highest primary stability, followed by ZPs and PPs under both transverse (PP: 0.36 ± 0.08 kN/mm vs. MP: 1.30 ± 0.31 kN/mm, *p* < .001; vs. ZP: 0.87 ± 0.33 kN/mm, *p* = .012) and axial loading (PP: 0.43 ± 0.10 kN/mm vs. MP: 1.25 ± 0.31 kN/mm, *p* < .001; vs. ZP: 0.77 ± 0.18 kN/mm, *p* = .035). Fracture displacement after 1,000 cycles was lower with MPs and ZPs than PPs (PP: 0.038 ± 0.009 mm vs. MP: 0.013 ± 0.003 mm, *p* < .001; vs. ZP: 0.022 ± 0.007 mm, *p* = .003). MPs (282 ± 26 N) showed the highest load-to-failure at 2 mm dislocation, followed by ZPs (261 ± 38 N) and PPs (215 ± 53 N) (PP vs. MP: *p* = .032; PP vs. ZP: *p* = .164; MP vs. ZP *p* = .650).

**Conclusion:**

In this biomechanical model of Mason type II radial head fractures, biodegradable magnesium and zinc pins demonstrated superior primary stability and load-bearing capacity compared to polylactide implants. MP showed the highest stiffness and lowest fracture displacement, while ZP achieved comparable performance in fracture stabilization. These findings suggest that zinc-based implants could offer a clinically valuable alternative for radial head fracture fixation, potentially reducing complications seen with the other implants. Further in-vivo, cadaveric, and clinical studies are necessary to confirm long-term outcomes and biological integration.

**Level of evidence:**

Basic Science Study.

## Introduction

Radial head fractures are among the most common elbow injuries in adults, typically resulting from falls on an outstretched forearm [1]. By varying from non-displaced two-part to complex multifragmentary fractures, they account for approximately one-third of elbow joint fractures and 1–4% of overall fractures in adults [1, 2]. Notably, radial head fractures of increasing severity are often associated with additional injuries and play a critical role in overall elbow joint stability and load transmission [3–6]. Therefore, proper management is essential to restore joint function and prevent long-term complications such as stiffness, instability, or osteoarthritis [7]. Minimally displaced radial head fractures (Mason type I) are typically managed non-operatively, yielding excellent long-term outcomes [8–10]. The optimal treatment for Mason type II fractures remains controversial [11, 12]. However, evidence suggests that primary open reduction and internal fixation (ORIF), especially in patients with high functional demands, is achieving good to excellent results [12, 13]. In fractures with associated elbow joint dislocation (Mason Type IV), even if simple, surgical fixation is typically required, and resection is contraindicated due to the risk of consecutive instability [14]. While all osteosyntheses should allow for early functional rehabilitation and pave the way to recover full range of motion, the used implants can vary from mini-fragment screws over osteosynthesis plates to small pins, depending on the individual fracture pattern and surgeons’ preference [15, 16]. Traditional fixation methods commonly employ metal implants such as titanium or stainless-steel screws and plates [17–19]. However, the disparity in elastic modulus between these materials (e.g., steel or titanium) and the native bone may contribute to stress shielding, implant-related irritation, aseptic loosening, and secondary need for implant removal [20]. These secondary surgeries carry additional risks, including stiffness and soft tissue complications, which ultimately can lead to further restrictions in movement due to arthrofibrosis [21].

In recent years, biodegradable implants have gained rising interest and offer promising alternatives by eliminating the need for implant removal and potentially reducing the risk of implant-associated complications due to their higher biocompatibility and less cartilage damage at the fracture site [22–25]. Polymer-based implants like polylactide pins are well-established but limited by their inferior mechanical strength and tendency for higher rates of secondary loss of reduction compared to metal implants [25, 26]. Magnesium-based implants provide higher stability and favorable osseointegration; however, concerns persist regarding their degradation behavior, particularly due to hydrogen gas evolution, which can create painful cavities within the surrounding tissues and inconsistent corrosion rates [23]. More recently, zinc-based alloys have emerged as a new class of metallic biodegradable implants, offering improved mechanical integrity, consistent degradation rates, and favorable biocompatibility [27, 28]. Although these biomechanical studies have been promising – demonstrating that the mechanical properties of zinc implants are comparable to those of magnesium implants – no biomechanical studies to date have tested these implants in a validated fracture model. Therefore, the present study aims to biomechanically compare the performance of polylactide, magnesium, and zinc pin implants for the fixation of displaced radial head fractures using a Mason type II radial head fracture model that has been established and repeatedly applied in previous biomechanical studies [22, 25, 29].

## Materials and methods

**Fig. 1:**
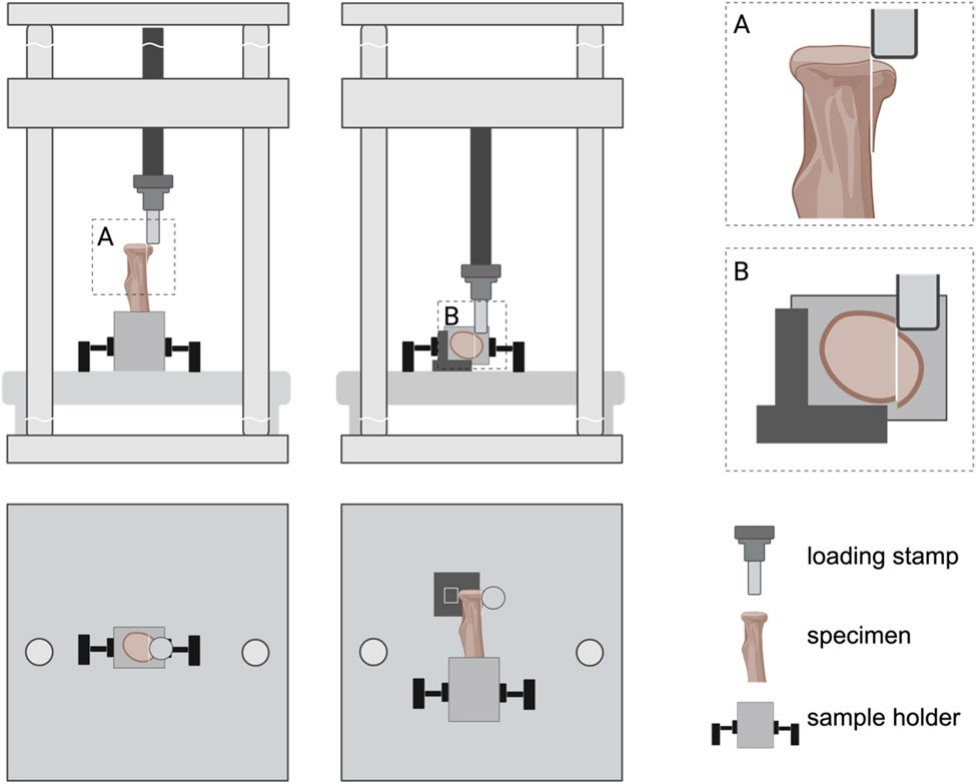
Illustration of experimental setup for biomechanical loading. Experimental Setup of axial (**A**) and transverse (**B**) loading illustrated from side- and top-view. Note that stamp pressure was applied to the fragment only, parallel to the fracture plane. The figure was created in BioRender. Maier, J. (2025) https://BioRender.com/uh65p4z

**Fig. 2:**
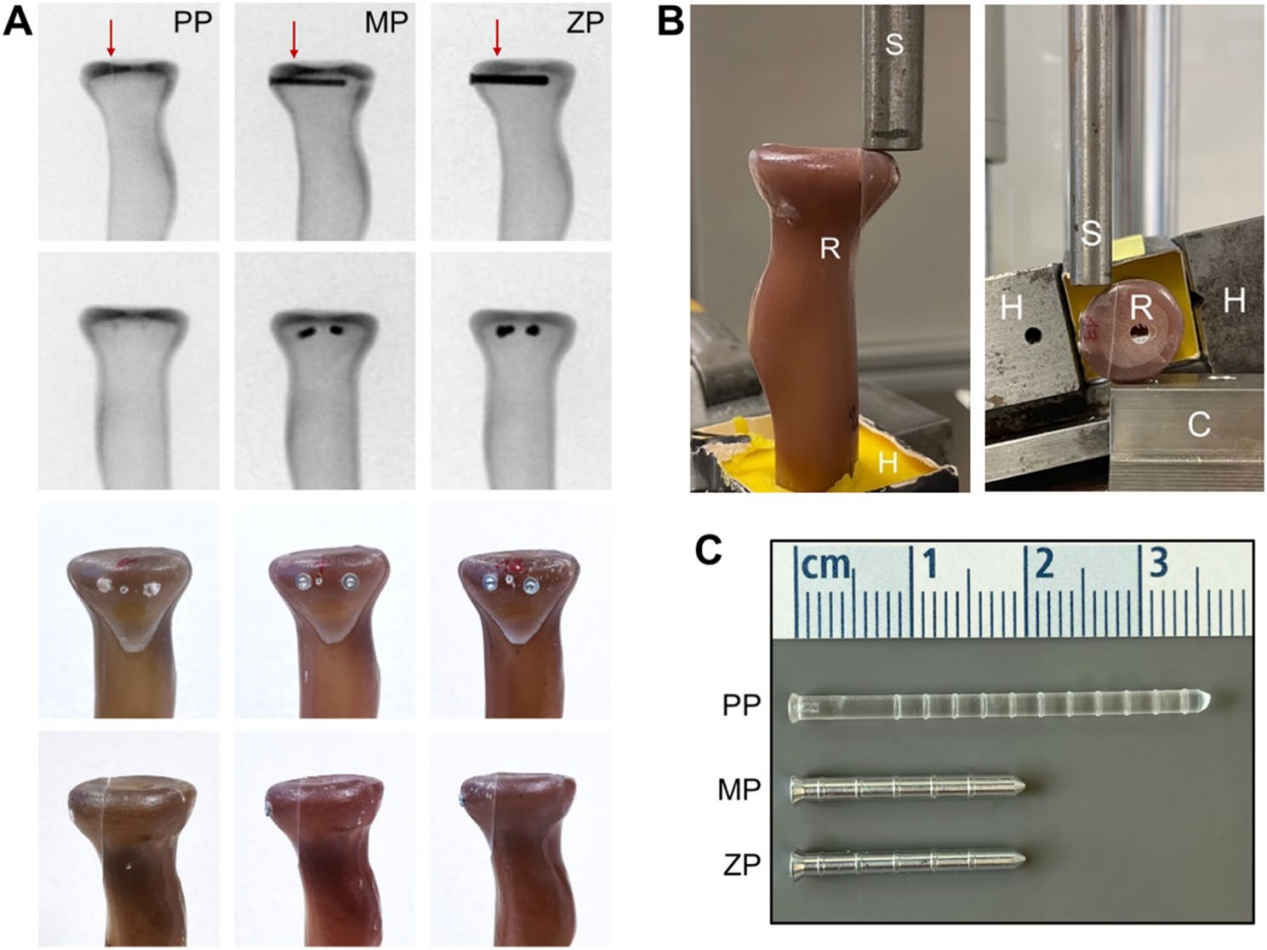
Representative samples, biomechanical loading setup, and osteosynthesis implants. **A**: Representative samples with red arrows indicating fracture lines. Plain radiography (X-rays) was used for control of fracture replacement and implant placement. **B**: Biomechanical testing of radius samples (R) mounted to a specific holding unit (H) under axial (left) and transverse (right) loading applied by the hydraulic pressure stamp (S). Note that stamp pressure was applied to the fragment only, parallel to the fracture plane. For transverse loading an additional counterpart (C) was used for sample fixation. **C**: Representative osteosynthesis implants from the manufacturer. Implants’ length was adjusted to a standardized length of 20 mm for all samples before use. PP, Polylactide Pin; MP, Magnesium Pin; ZP, Zinc Pin

Biomechanical testing was performed by using eighteen biomechanically validated synthetic radii (Radius absolute™ 4th Gen. 17 PCF Solid Foam Core Large, Sawbones^®^ Europe AG, Malmo, Sweden). Type Mason II radial head fractures were conducted by a standardized procedure as described previously by our biomechanics research group [22, 25]. The exact fracture plane was determined before sawing, and an adjustable specimen clamp was used to guarantee satisfying osteotomy precision. Radial head fracture fragments measured one-third of the radial head’s longest diameter, including the safe zone of the radial head circumference (Figs. 1 and 2). Fractures were ending tangentially to the radial shaft, ensuring that fragments had no bony support during axial or transversal loading. The samples (*N* = 18) were divided into three subgroups (*n* = 6) for fracture reduction and fixation by either two 2.0 × 20 mm magnesium pins (MP) (LiMedion GmbH, Mannheim, Germany), zinc pins (LiMedion GmbH, Mannheim, Germany), or polylactide pins (PP) (PolyPIN^®^; Biovision, Ilmenau, Germany), respectively (Fig. 2). The length of polylactide pins (PP) was manually adjusted to the same length as the other pins (20 mm) before use. Magnesium pins (MP) were composed of WE43, as used in previous studies [30, 31]. Zinc pins (ZP) were composed of a zinc-silver alloy containing 3.3 wt% silver, biomechanically validated in a previous study [27]. All implants were implanted according to the manufacturer’s instructions. The pins were implanted parallel to the articular joint’s surface. The final osteosyntheses were radiologically controlled in two planes (Fig. 2). Constructs were shortened and embedded into standardized carton cuboids in polymethylmethacrylate (PMME Technovit 3040; Heraeus Kulzer GmbH, Wehrheim, Germany), leaving a free bone stock of approximately 5 cm to ensure adequate mounting into the testing machine. Implant testing was carried out using the servo-hydraulic testing machine Amsler HC10 (Zwick/Roell, Ulm, Germany). A cylindrical stamp applied loading forces, axial and transverse to the fracture line, as described previously, with a maximum force of 50 Newtons (≈ 5.10 kg-force [kgf]) [22, 25]. This load level approximates the forces experienced by the radial head during passive range-of-motion exercises and light daily activities, which are typically permitted in the initial postoperative period. Axial loading was tested by placing the radial shaft vertically to the testing machine’s load frame using a mounting clamp (Figs. 1 and 2). In transverse loading, the samples were placed in their cross-axis, with a metal block as a counterpart supporting the radial head except for the fragment (Figs. 1 and 2). In both settings, it was ensured that the loading was parallel to the fracture plane, and any support of the fragment was prevented. First, transverse cyclic loading was performed by 10 sinusoidal load changes between 15 and 50 N at 0.1 Hz, followed by long-cycle testing under axial loading with sinusoidal load changes at 0.1 Hz between 15 and 50 N for a total of 1,000 cycles. Subsequently, load-to-failure testing was performed by continuously increasing the axial load (2 N per second) until ≥ 2 mm fracture displacement and subsequent construct failure under maximum load occurred. The real-time fracture displacement (mm) and loading forces (N) were constantly monitored by the testing machine. Final measurements and outcome parameters included primary stability in terms of construct stiffness (kN/mm), construct loosening (amplitude of fracture displacement in mm) after 1,000 cycles of axial load, and load-to-failure (N) until fracture displacement ≥ 2 mm and complete construct failure.

Statistical analysis and data visualization were conducted using R software (version 4.2.3). Normality and homogeneity of the datasets were assessed using the Shapiro–Wilk test and Levene’s test, respectively. As the assumptions of normality and homogeneity were met, one-way analysis of variance (ANOVA) was performed for each outcome measure to assess overall group differences (PP, MP, ZP). Post hoc comparisons were conducted using Tukey’s honestly significant difference (HSD) test to identify pairwise differences between groups (e.g., PP vs. MP, PP vs. ZP). Statistical significance was defined as *p* < .05.

## Results

Under transverse and axial loading (Figs. 1 and 2) between 15 and 50 N at 0.1 Hz, all three groups showed statistically significant differences in primary stability. The highest stiffness was recorded within the group of MP constructs, followed by ZP osteosyntheses, and fixation with PP showing the lowest stability both under transverse (PP: 0.36 ± 0.08 kN/mm vs. MP: 1.30 ± 0.31 kN/mm, *p* < .001; vs. ZP: 0.87 ± 0.33 kN/mm, *p* = .012; MP vs. ZP: *p* = .033), and axial loading (PP: 0.43 ± 0.10 kN/mm vs. MP: 1.25 ± 0.31 kN/mm, *p* = .001; vs. ZP: 0.77 ± 0.18 kN/mm, *p* = .035; MP vs. ZP: *p* = .004) (Table 1).

Fracture displacement was tested after 1,000 cycles of axial loading, showing the least fracture displacement in MP and ZP constructs compared to osteosynthesis with polylactide pins (PP: 0.038 ± 0.009 mm vs. MP: 0.013 ± 0.003 mm, *p* < .001; vs. ZP: 0.022 ± 0.007 mm; *p* = .003). Groupwise comparison revealed significantly lower fracture displacement of both MP and ZP constructs compared to PP osteosyntheses (Table 1). Although primary stability was higher with fixation using MP, no statistically significant difference in fracture displacement was observed between MP and ZP constructs (*p* = .062).

**Table 1:** Overall results of Biomechanical testing

Variable	Mean	SD	Median	Adj. *p*-value	
*Stability transv. (kN/mm)*					
PP	0.360	0.081	0.351	PP vs. MP	< 0.001
MP	1.300	0.308	1.374	PP vs. ZP	0.012
ZP	0.870	0.330	0.854	MP vs. ZP	0.033
*Stability axial (kN/mm)*					
PP	0.426	0.096	0.419	PP vs. MP	< 0.001
MP	1.254	0.312	1.174	PP vs. ZP	0.035
ZP	0.771	0.180	0.790	MP vs. ZP	0.004
*Displacement after 1,000 cycles (mm)*					
PP	0.038	0.009	0.035	PP vs. MP	< 0.001
MP	0.013	0.003	0.014	PP vs. ZP	0.003
ZP	0.022	0.007	0.021	MP vs. ZP	0.062
*Load-to-failure (N) at 2 mm displacement*					
PP	215	53	207	PP vs. MP	0.032
MP	282	26	288	PP vs. ZP	0.164
ZP	261	38	262	MP vs. ZP	0.650
*Max. Load (N) until construct failure*					
PP	253	48	249	PP vs. MP	0.010
MP	341	17	342	PP vs. ZP	0.041
ZP	322	58	329	MP vs. ZP	0.755

Clinically relevant fracture displacement in load-to-failure testing was defined as ≥ 2 mm. SD, standard deviation; kN, kilonewton; N, newton; mm, millimeter; PP, Polylactide Pin; MP, Magnesium Pin; ZP, Zinc Pin

**Fig. 3:**
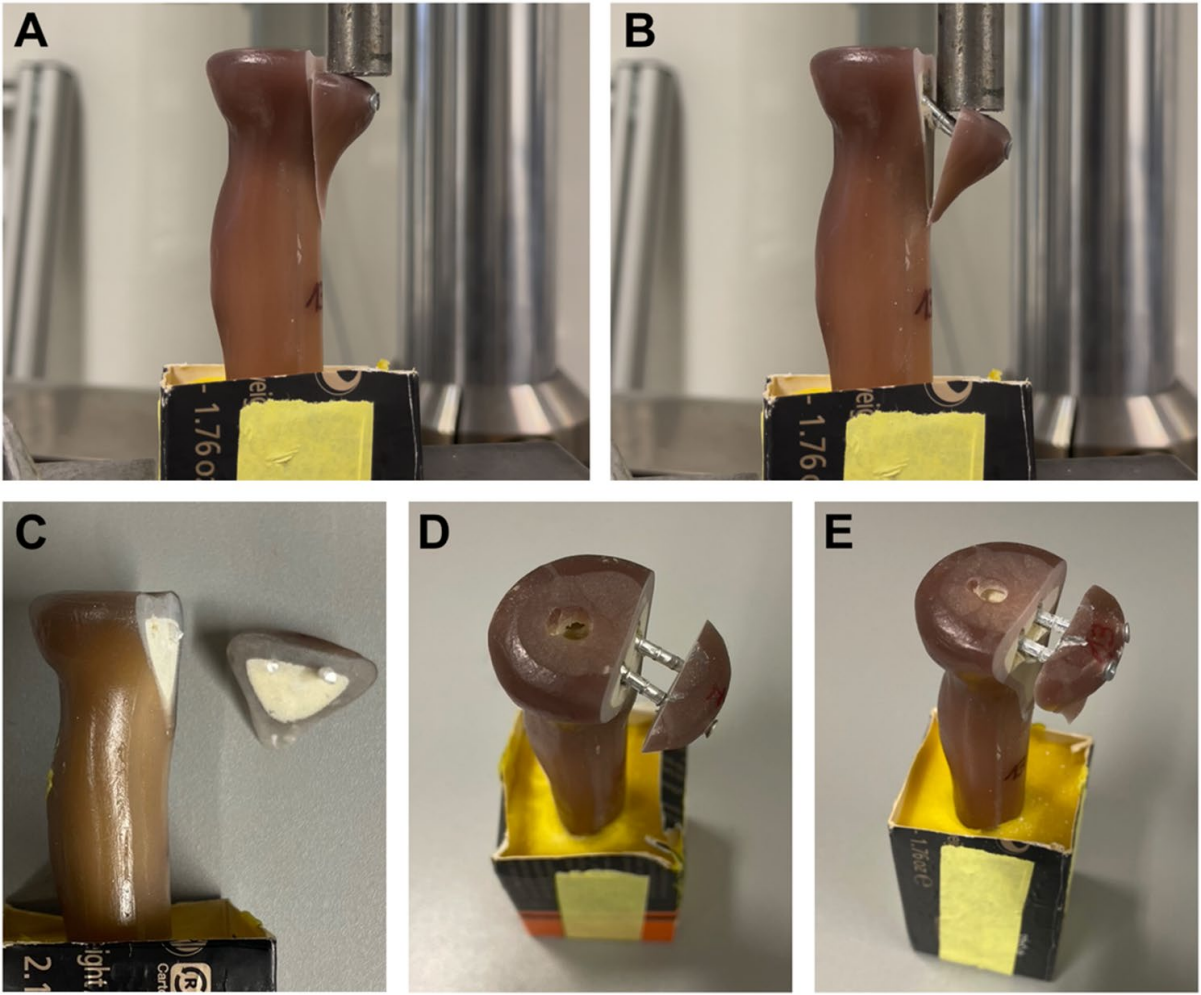
Load-to-failure Measurements under axial loading. Load-to-failure was tested under axial loading with increasing loading force of 2 N per second until complete construct failure (**B**). Relevant fracture displacement (**A**) was defined at ≥ 2 mm. Representative samples after load-to-failure testing with construct failure of Polylactide Pins (**C**), Magnesium Pins (**D**), and Zinc Pins (**E**). Note that only in Polylactide Pins (**C**) implant breakage occurred

Load-to-failure testing (Fig. 3) with increasing load (2 N per second) revealed the highest failure loads in MP constructs (282 ± 26 N) at 2 mm fracture displacement. These results were significantly higher compared to polylactide pins (PP: 2 mm: 215 ± 53 N, *p* = .032). However, in comparison to zinc implants, there was no significant difference (ZP: 2 mm: 261 ± 38 N, *p* = .650). In the final comparison between polylactide and zinc implants, there was a clear trend suggesting superior load-to-failure performance of the zinc (ZP) constructs; however, the differences did not reach statistical significance at 2 mm fracture displacement (*p* = .164) (Table 1). When analyzing the maximum loads required to cause complete construct failure, both magnesium and zinc implants performed significantly better than polylactide pins (PP: 253 ± 48 N vs. MP: 341 ± 17 N, *p* = .010; vs. ZP: 322 ± 58 N, *p* = .041), further underscoring their superior biomechanical properties (Table 1). The comparison between MP and ZP constructs revealed no statistically significant difference in construct failure under maximum loads (*p* = .755). Notably, only polylactide pins exhibited implant breakage under maximum loading forces, whereas the zinc and magnesium pins demonstrated bending of the implants, accompanied by substantial fracture displacement (Fig. 3).

## Discussion

The optimal fixation strategy for Mason type II radial head fractures remains a topic of ongoing debate, particularly in the context of implant material. This study aimed to evaluate the biomechanical properties of three different biodegradable pin implants – magnesium (MP), zinc (ZP), and polylactide (PP) – in a standardized Mason type II radial head fracture model. In the present study, different numbers of loading cycles were applied for the two test modes to reflect their distinct clinical relevance. Tangential (transverse) loading was limited to 10 cycles, as such forces mainly represent initial micromotion at the articular surface during reduction and early postoperative handling. This test mode primarily served to assess the stability of the anatomic reduction and the sufficiency of the restored radial head circumference. Once this stability is ensured, subsequent repetitive loadings to an anterior fragment occur mainly during elbow flexion, which is more appropriately simulated by axial loading. Therefore, axial loading was extended to 1,000 cycles to mimic the repeated joint forces encountered during early rehabilitation and daily activities following radial head fracture fixation. Our findings demonstrate a clear biomechanical advantage of metallic biodegradable implants (MP and ZP) over polymer-based alternatives (PP), reinforcing previous concerns about the limited mechanical capacity of polylactide in high-demand anatomical regions [25, 26].

Under both axial and transverse cyclic loading, magnesium implants consistently showed the highest stiffness, followed by zinc and polylactide. These results align with prior studies, which have indicated the superior load-sharing properties of magnesium-based implants due to their higher modulus of elasticity and strength, approaching that of cortical bone [22, 23, 32, 33]. Zinc implants, while slightly less stiff than magnesium, performed significantly better than polylactide, suggesting that their mechanical profile may indeed offer a clinically viable alternative.

Fracture displacement after cyclic loading further confirmed the mechanical limitations of PP constructs, which demonstrated significantly greater displacement compared to both MP and ZP constructs. Notably, there was no significant difference in fracture displacement after cyclic loading between the metallic MP and ZP constructs, although primary stability was slightly higher with fixation using MP, suggesting comparable efficacy of ZP in fracture stabilization.

The overall higher primary stability and significantly reduced early fracture displacement of biodegradable metallic constructs may allow for earlier functional postoperative care, which is typically more restricted following polypin osteosynthesis [26]. However, clinical outcome parameters such as reduced posttraumatic degeneration, fracture-related pain, or long-term secondary loss of reduction must be evaluated in prospective clinical trials, as the experimental setup of the present study could not address them.

Articular steps ≥ 2 mm are an ongoing subject to treatment discussions [11, 12], whereas isolated injuries with articular steps < 2 mm (Mason type I) are usually treated non-operatively with good clinical results [8]. Hence, fracture displacement ≥ 2 mm was determined as the relevant cutoff in the axial load-to-failure testing as reported in previous biomechanical studies [22, 25, 29, 34]. Load-to-failure testing revealed no significant difference between MP and ZP implants at 2 mm fracture displacement, underscoring the comparable performance of these two metallic biodegradable materials. These results are in line with previous studies evaluating the overall mechanical properties of zinc pins [27, 28]. Notably, the maximum load capacity was significantly higher for both MP and ZP compared to PP. Moreover, only PP implants exhibited brittle failure by breakage of implants, whereas MP and ZP constructs showed ductile deformation, a potentially favorable failure mode in clinical settings.

The surgical technique for implanting the tested bioresorbable pins is comparable, as all implants are inserted by impaction following the creation of a single 2.0-mm drill hole per pin. Due to their nearly identical shape and adjustable length, variables such as exposure, surgical approach, or surgeons’ experience are unlikely to influence primary outcomes or reduction quality. Additionally, all implants are suitable for use in small fragments and can be placed beyond the safe zone, offering advantages over bulkier fixation devices as described previously [22].

Notably, several magnesium alloys are available for use in biomedical implants, each exhibiting distinct corrosion characteristics, as recently elaborated in a review by Wei and Gao [35]. In our study, magnesium pins composed of the WE43 alloy were specifically selected because this alloy is frequently used in CE-certified implants for clinical trauma applications within Europe and has been evaluated in multiple biomechanical and clinical investigations [36–38]. Overall, the biocompatibility [39], stable degradation profile [27, 28], and comparable mechanical performance of the tested zinc pin implants place it as a compelling alternative to magnesium pins, especially given concerns about hydrogen gas formation and unpredictable corrosion of magnesium implants [23, 27]. These results support further investigation into zinc-based orthopedic implants for the fixation of joint fractures.

In addition to their potential in radial head fracture fixation, biodegradable metallic implants composed of magnesium and zinc are being increasingly explored in other anatomical regions and clinical applications. For example, magnesium interference screws have demonstrated high fixation ability and workability without material failure in anterior cruciate ligament (ACL) reconstruction [40]. Similarly, zinc-based implants are under investigation for use in various orthopedic and trauma indications, where their favorable balance of mechanical3 strength, bone integration ability, antibacterial properties, and controlled degradation profile may offer advantages [41–43]. These findings underscore the broader translational potential of biodegradable metallic implants across different anatomical districts in orthopedic and trauma surgery, extending beyond the upper extremity.

From a resource perspective, magnesium is globally abundant, making it an attractive candidate for the large-scale development of cost-effective implants [44, 45]. In contrast, zinc is less widely available, and silver is comparatively scarce and costly, which may limit the widespread applicability of zinc-silver alloys in routine orthopedic implant production. These disparities in availability and cost are important factors to consider when evaluating the long-term clinical potential and sustainability of biodegradable implants. Nevertheless, zinc-based implants remain in the earlier stages of development, and their widespread clinical application will depend on further advancement of manufacturing processes and comprehensive in vivo evaluations [39, 46].

This study has several limitations inherent to its in vitro design. As described previously by our research group, the absence of soft tissues such as the joint capsule and ligaments may affect the generalizability of the findings, as these structures contribute significantly to the overall elbow joint stability [5, 22]. Additionally, the isometric load application did not account for dynamic joint movements, including shear forces, bending moments, and varus-valgus stresses, which would more closely replicate physiological conditions [22]. Further, the resorption behavior of biodegradable implants is influenced by biological factors such as vascular supply and cancellous bone contact, which could not be assessed by our model as described previously [22]. However, the use of fourth-generation composite bone models – validated to closely mimic the biomechanical properties of human bone under loading forces – allowed for standardized specimen comparison and uniformity that cannot be achieved in cadaveric studies [47]. Lastly, the small group size (*n* = 6) may have limited the detection of subtle differences; however, it is consistent with sample sizes used in comparable biomechanical investigations [48–52].

## Conclusion

In this biomechanical model of Mason type II radial head fractures, biodegradable magnesium and zinc pins demonstrated superior primary stability and load-bearing capacity compared to polylactide implants. Magnesium pins showed the highest stiffness and lowest fracture displacement, though zinc pins offered comparable overall performance without implant failure. These findings suggest that zinc-based implants may provide a promising, biomechanically robust alternative for radial head fracture fixation, potentially mitigating complications associated with magnesium or polylactide implants. Future cadaveric, in-vivo, and clinical studies are warranted to evaluate long-term outcomes and biological integration of these novel implants.

## Data Availability

The datasets generated and/or analyzed during the current study are not publicly available but are available from the corresponding author upon reasonable request.
